# Single-cell analysis reveals sexually dimorphic repertoires of Interferon-γ and IL-17A producing T cells in salivary glands of Sjögren’s syndrome mice

**DOI:** 10.1038/s41598-017-12627-6

**Published:** 2017-10-02

**Authors:** Arun Wanchoo, Alexandria Voigt, Sukesh Sukumaran, Carol M. Stewart, Indraneel Bhattacharya, Cuong Q. Nguyen

**Affiliations:** 1 Department of Infectious Diseases and Pathology, College of Veterinary Medicine, University of Florida, Gainesville Florida, USA; 2 Rheumatology Section, University of Arkansas for Medical Sciences, Arkansas Children’s Hospital, Little Rock Arkansas, USA; 3 Department of Oral and Maxillofacial Diagnostic Sciences, Gainesville Florida, USA; 4 Department of Oral Biology, Gainesville Florida, USA; 5 Center of Orphaned Autoimmune Diseases, University of Florida, Gainesville Florida, USA

## Abstract

The development of Sjögren’s syndrome (SjS) is a dynamic and temporal process with a female predilection. Following the initial influx of immune cells, T cell clusters develop, accelerating the pathology in the salivary glands. Proinflammatory cytokines, IFN-γ and IL-17A, produced by T cells contribute synergistically to the disease. In this study, we examined the sexual dimorphism in cellular infiltrates of the salivary glands by using functional single-cell microengraving analysis. Using high-throughput sequencing, we investigated the clonal diversity of the T cell receptors (TCRs) of infiltrating IFN-γ and IL-17A-producing T cells in male and female SjS-susceptible (SjS^s^) C57BL/6.NOD-*Aec1Aec2* mice. There were elevated frequencies of IFN-γ and IL-17A-producing effector T cell populations in female SjS^S^ mice compared to male SjS^S^ mice. MEME analysis shows high frequency and unique, sexually dimorphic motifs in the TCR hypervariable regions in the SjS^S^ mice. Male mice selected for TRAV8/TRAJ52 (CATDLNTGANTGKLTFG) TCR genes in Th1 cells and TRBV16/(TRBD1/2)TRBJ1-7 (CGGKRRLESIFR) in Th1 and Th17 cells. Female SjS^S^ mice selected for TRAV8/TRAJ52 (CATDLNTGANTGKLTFG), TRAV13D-2/TRAJ23 (CVYLEHHFE), and TRBV23/(TRBD2)TRBJ2-2 (CRKLHSCATCALNFL) in Th1 cells. These findings suggest that there is an elevated prevalence of pathogenic effector T cells in the glands with a sexually dimorphic selection bias of TCR repertoires.

## Introduction

Sjögren’s syndrome (SjS) is an autoimmune disorder characterized by the inflammation of secretory glands, namely the lacrimal and salivary glands (SG) leading to keratoconjunctivitis and xerostomia i.e. dry eyes and dry mouth^1^. Immunoglobulins, including autoantibodies against ribonuclear proteins such as SSA/Ro and SSB/La, are highly elevated in SjS patients. The disease progression is marked by the unregulated infiltration of lymphocytes in which some foci organize into germinal center-like formations in the glands causing periductal aggregates, inflammation, and apoptosis; this leads to gland dysfunction and loss of tear and saliva production^2–4^. CD4^+^ T helper cells (Th) expressing the αβ T cell receptor (TCR) dominate the infiltrates^5–8^. SjS presents a signature of type 2 interferon, Interferon-γ (IFN-γ) as well as Interleukin (IL)-17A likely produced by Th1 and Th17 cells respectively^9–11^.

Human and animal models of SjS exhibit high levels of IFN-γ and IFN-responsive factors. The upregulation of the IFN pathway induces the activation of macrophages, natural killer (NK) cells, and CD8^+^ T cells. It also induces vascular adhesion molecule-1 (VCAM-1), L-selectin, lymphocyte function-associated antigen-1 (LFA-1), and other molecules that can trigger the homing of immune cells to the glands^12^. Previous data indicated that organogenesis in the SG is retarded in the SjS-susceptible (SjS^S^) NOD (non-obese diabetic) model and ablation of *Ifnγ* resulted in a return to normal gland development^13^. It is very likely that IFN-γ plays a role in early disease development as well as the symptoms by attracting invasive lymphocytes as well as hindering gland development, which in turn exacerbates glandular dysfunction. In addition to IFN-γ producing Th1 cells, we, and others, have shown that IL-17A producing Th17 cells contribute profoundly to the disease pathogenesis^10,14–16^. L-17 is expressed as one of six isotypes, two of which are defining to Th17 cells (IL-17A and IL-17F) with IL-17A being the more prevalent isotype^17^. In addition to production of IL-17, which recruits neutrophils, Th17 cells produces IL-21 and IL-22, which regulates B cell differentiation and immunoglobulin isotype switching, and induces proliferation in a STAT3-dependent manner, respectively^18^. Our recent studies have indicated that IL-17A plays a critical role in the strong sexual dimorphism in the SjS^S^ mouse model where it affects sialadenitis, plasma cells, and germinal center B cell populations, moreso in females^19,20^. Furthermore, because IL-17’s roles in inducing isotypic switching, recruiting neutrophils, and inducing proliferation, it strongly suggests that Th17 cells may have an intrinsic role in both disease development and progression.

The essential role of Th1 and Th17 cells at the specific stages of the autoimmune process is widely studied. In addition to the Th subsets, cytotoxic T cells expressing IFN-γ or IL-17A (Tc1 and Tc17 respectively) have been identified as propagating autoimmunity in other diseases^21–23^. In context of the major histocompatibility complex (MHC) expressed on antigen presenting cells, the effector T cell recognizes a peptide antigen in a specific interaction via the TCR, a heterodimeric membrane protein that comes in two forms, αβ and γδ, the former of which is present in 70% of T cells infiltrating the SG^24^. The recognition of cognate antigen triggers activation, cytokine secretion, and proliferation^25^. During the immune response, antigen-specific interactions lead to proliferation of only reactive T cells, this, in turn, leads to clonotypic restriction and loss in diversity^26–28^. Diversity of the TCR is generated from the unique pairing of variable (V) and joining (J) gene segments for the α chain or V, diversity (D), and J in the case of the β chain, which generates on the order of 1014 possible combinations^29,30^. Each chain possesses a hypervariable region or complementarity determining region 3 (CDR3), which contains amino acids responsible for interaction with the antigen. Diversity is skewed toward CDR3 in αβ TCRs as opposed to CDR1 and CDR2 regions, which contact the cognate MHC. In response to antigen interaction, non-template nucleotides (randomly added in a terminal deoxynucleotidyl transferase dependent manner), N in the α chain or NDN in the β chain, are inserted between the V and J gene segments during recombination which leads to a change in the CDR3 region length^31,32^. In the TCR repertoire, each clone is represented by a single conserved TCR sequence; a robust T cell response is characterized by a reduction of heterogeneity and restriction of epitopes of antigen presentation, i.e. monoclonal expansions of antigen experienced T cells.

In this study, we investigated the comprehensive TCR repertoires of individual infiltrating T cells in the SGs by analyzing the TCRαβ transcripts of CD4^+^ and CD8^+^ T cells that produce either IFN-γ or IL-17A in a SjS^S^ animal model. Our results indicate that the TCR repertoire of the Th1 and Th17 cells in the SG of SjS^S^ mice exhibited reduced diversity in the CDR3 regions, which expressed predominantly TRAV8/TRAJ52 (Th1) and TRBV16/(TRBD1/2)TRBJ1-7 (Th1 and Th17) in male and female mice as well as TRAV13/TRAJ23 and TRBV23/(TRBD2)TRBJ2-2 (Th1) in female mice. These clones indicate antigen mediated maturation of effector T cells to a common autoantigen in the SjS^S^ mouse model. This helps elucidate the underlying initiation of the autoimmune cascade in SjS.

## Results

### Isolation of helper and cytotoxic T cells by microengraving shows an increase in IFN-γ and IL-17A producing cells in SjS^S^ glands

**Figure 1:**
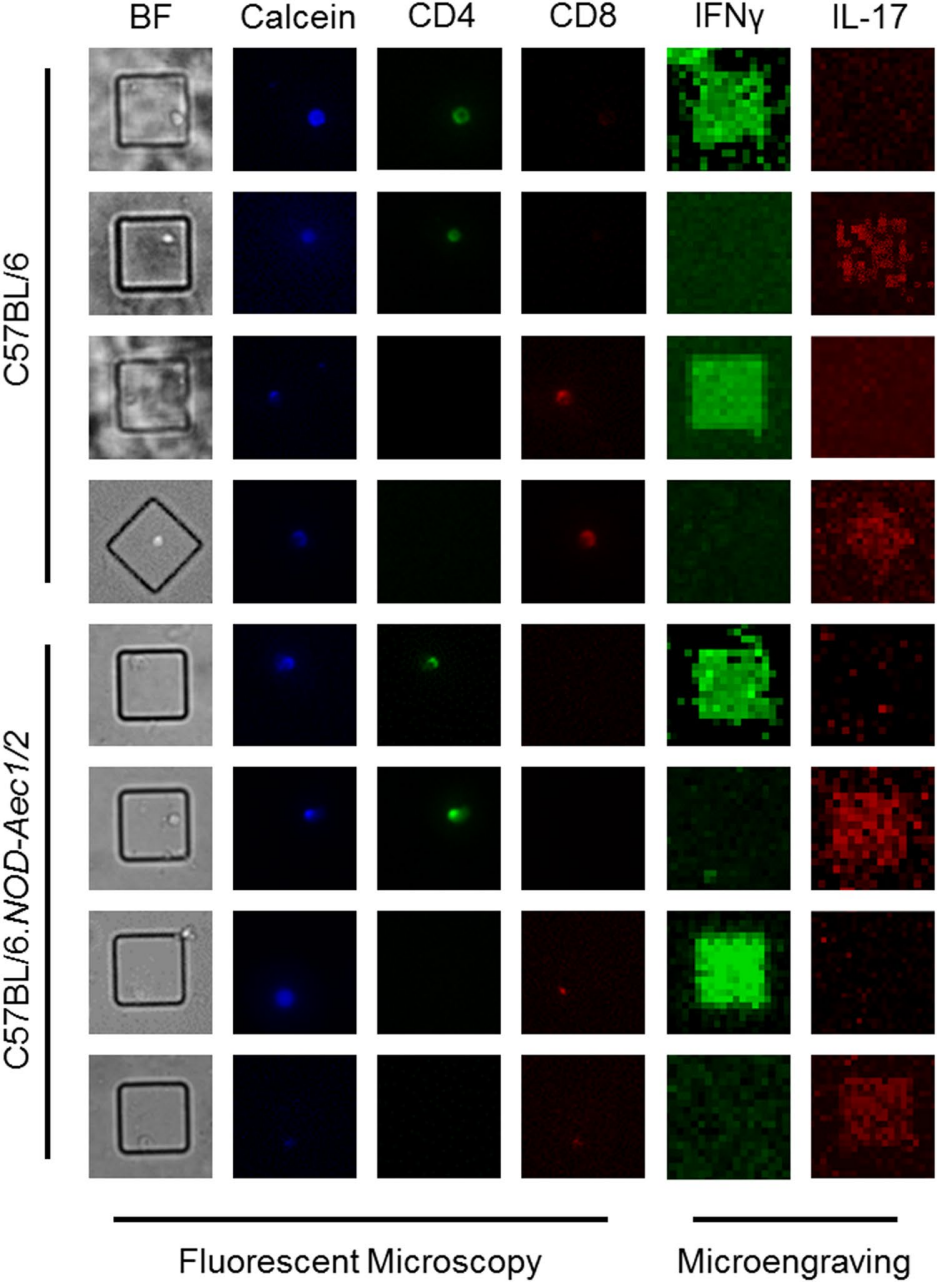
Microengraving of T lymphocytes isolated from murine salivary glands. Representative fluorescent microscopy coupled with microengraving of secreted cytokines from isolated T-lymphocytes. Fluorescent antibody staining was performed with anti-CD4-FITC (green), anti-CD8-PE (Red), and Calcein violet-405 (blue), a marker of viable cells. Secreted cytokines were captured during microengraving and stained with anti-IFN-γ (green) and anti-IL-17A (red).

**Figure 2:**
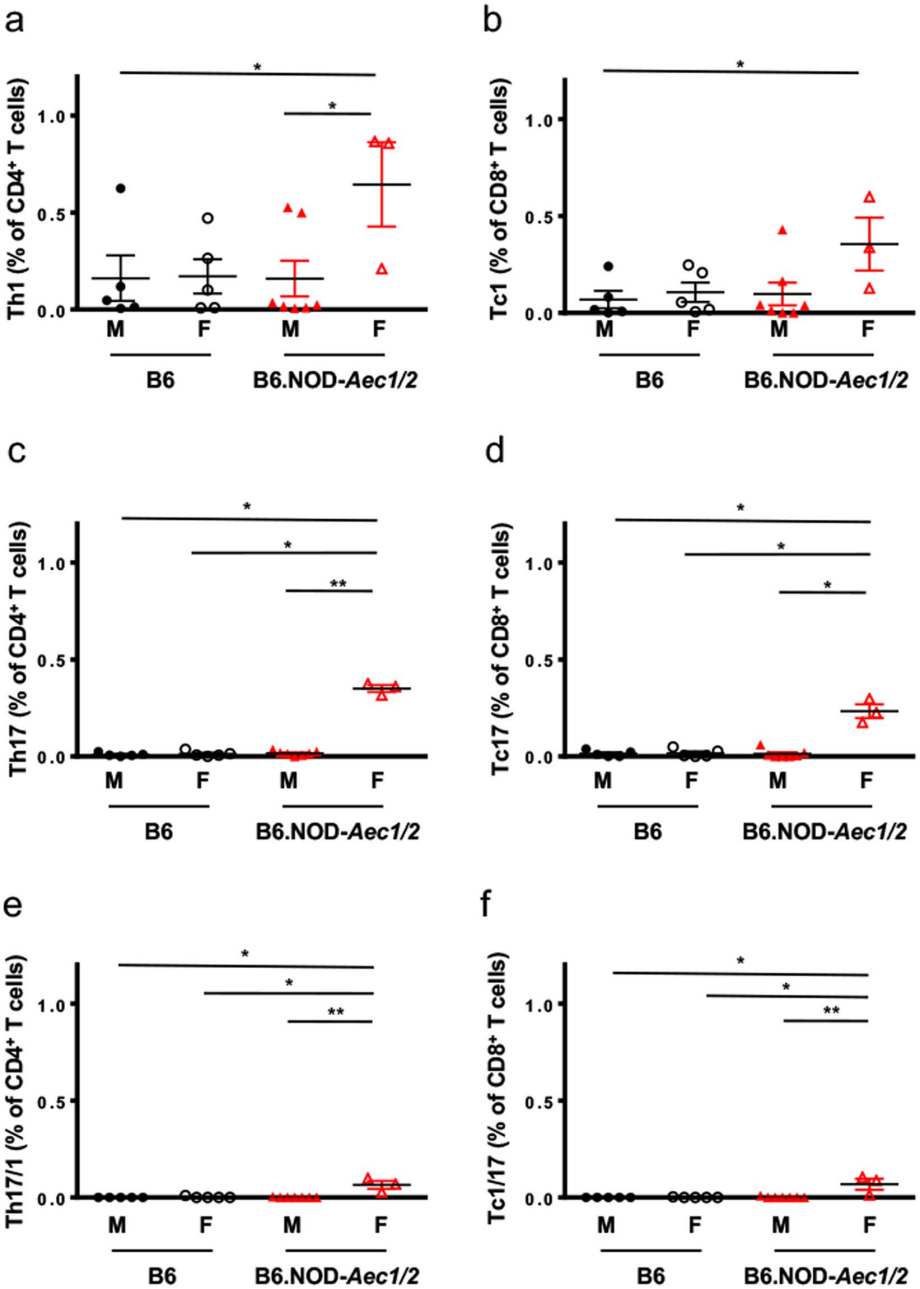
Microengraving shows greater infiltration by IL-17A and IFN-γ producing cells in the salivary glands of SjS^S^ mice. Quantification of T lymphocytes isolated from the major salivary gland of B6 male (●), B6 female (○), B6.NOD-*Aec1Aec2* male (), and B6.NOD-*Aec1Aec2* female () mice expressing (**a**) CD4^+^IFN-γ^+^, (**b**) CD8^+^IFN-γ^+^, (**c**) CD4^+^IL-17A^+^, (**d**) CD8^+^IL-17A^+^, (**e**) CD4^+^IFN-γ^+^IL-17A^+^, or (**f**) CD8^+^IFN-γ^+^IL-17A^+^. The frequency in percentage of was determined by using the percentage (multiplied by 100) of the total number of CD4^+^IFN-γ+ (Th1), CD4^+^IL-17A+ (Th17), CD8^+^IFN-γ+ (Tc1), CD8^+^ IL-17A+ (Tc17), CD4^+^IL-17A+IFN-γ+ (Th17/1), and CD8^+^IL-17A+IFN-γ+ (Tc1/17) cells from wells with single live cells among the total number of wells with single CD4^+^ or CD8^+^ cells. Group comparisons were made using a Kruskall-Wallis test with Dunn’s post hoc test. Statistical significance was defined as a p value < 0.05. All results are presented as means ± s.e.m. Significance was determined as *p < 0.05, **p < 0.01.

Both IFN-γ and IL-17A are essential for the development of the autoimmune cascade in both SjS^S^ mice and patients^13–15,20,33–35^. Utilizing single-cell microengraving analysis with functional live cell characterization, we sought to isolate and characterize T helper (Th) and cytotoxic T (Tc) cells producing IL-17A and/or IFN-γ in the SG of SjS^S^ mice. Stimulated lymphocytes were labeled and microengraved to identify the following subsets for isolation: CD4^+^IFN-γ+ (Th1), CD4^+^IL-17A+ (Th17), CD8^+^IFN-γ+ (Tc1), CD8^+^ IL-17A+ (Tc17), CD4^+^IL-17A+IFN-γ+ (Th17/1), and CD8^+^IL-17A+IFN-γ+ (Tc1/17) (Fig. 1). Due to the established sexually dimorphic function of IL-17A in SjS, it is pertinent to examine the phenotypic differences by sex in the mice. As expected, there were no significant differences between male and female B6 mice in regards to the frequencies of Th1, Tc1, Th17, Tc17, and Tc1/17 in the SG. However, female B6 mice showed an elevated, but statistically insignificant frequency of Th17/1 cells over their male counterparts. By comparison, the SjS^S^ mice had a more than two-fold increase of infiltrating Th1, Tc1, Th17, Tc17, Th17/1, and Tc1/17 cells when compared to B6 mice (Fig. 2). The single-cell microengraving analysis did not alter the T cell subsets when compared to direct *ex-vivo* staining by flow cytometry (Supplementary Fig. S1 **)**. Interestingly, when the sexes were compared, it became apparent that there was a significant increase in these cell populations in female over male SjS^S^ mice. As presented in Table 1 and Fig. 2, female SjS^S^ B6.NOD-*Aec1/2* exhibited 2- and 18-fold increases of Th17 and Tc17 (0.350 versus 0.161, and 0.233 versus 0.013, respectively) in comparison to males; there was also an approximately 4- and 3-fold increase in Th1 and Tc1 cells, respectively, in SjS^S^ females versus males. Unexpectedly, there was a marked increase in the Th17-IFN-γ (65-fold) and Tc1-IL-17A (68-fold) populations in female SjS^S^ mice, compared to males. This suggests that the infiltrating T cells in the SG of the SjS^S^ mice are comprised of higher frequencies of effector T cells, particularly Th1, Tc1, Th17, Tc17, Th17-IFNγ, and Tc1-IL-17A cells. More importantly, female SjS^S^ B6.NOD-*Aec1/2* showed a significant increase in all the effector T cells compared to their male counterparts. These data clearly indicate that there is a significant infiltration of effector T cells in SjS^S^ B6.NOD-*Aec1/2* mice when compared to B6 mice, the most significant of which are those that produce IL-17A. Furthermore, these cell types are differentially detectable in males and females of B6.NOD*-Aec1/2* mice.

**Table 1:** Frequency of IFN-γ and IL-17A producing T cells in the salivary glands of male and female mice.

	B6M	B6F	B6.NOD-*Aec1/2 *M	B6.NOD-*Aec1/2 *F
Th1	0.161 ± 0.117	0.171 ± 0.088	0.159 ± 0.092	0.644 ± 0.217 (4x)^#^
Th17	0.009 ± 0.004	0.013 ± 0.001	0.161 ± 0.004	0.350 ± 0.018 (2x)^#^
Th17/1	0.000 ± 0.000	0.002 ± 0.002	0.001 ± 0.001	0.065 ± 0.021^†^ (65x)^#^
Tc1	0.069 ± 0.045	0.107 ± 0.050	0.098 ± 0.059	0.355 ± 0.136 (3x)^#^
Tc17	0.014 ± 0.007	0.017 ± 0.009	0.013 ± 0.008	0.233 ± 0.035 (18x)^#^
Tc1/17	0.000 ± 0.000	0.001 ± 0.001	0.001 ± 0.001	0.068 ± 0.028^†^ (68x)^#^

Summary of means with ± s.e.m. of infiltrating T cells. Mean is defined as the percentage of Th/CD4^+^ T cells or Tc/CD8^+^ T cells. Statistics were performed using a Kruskall-Wallis test with Dunn’s post hoc test. ^#^Fold-change between female and male B6.NOD-*Aec1/2*. ^†^Denotes significance of p < 0.05 or less between all other data sets in a row.

### Loss of normal distribution in V/J pairing is associated with the SjS^S^ B6.NOD-*Aec1*/*2* mice

To explore the differences between individual effector T cells in the SG of males and females, functional live CD4^+^ and CD8^+^ T cells producing IFN-γ or IL-17A were examined for TCR gene rearrangement. After microengraving, each live cell that expressed IFN-γ or IL-17A, was isolated, then reverse transcription nested PCR was performed with two pairs of primers used in sequential reactions to target the amplification of the hypervariable, CDR3 region of the *tcra* and *tcrb* transcripts. Sequences were aligned to the International ImMunoGeneTics Information System (IMGT) using the IgBLAST tool to determine the V and J (and D) gene. Of the sequences recovered from B6 mice, 72% amplified the TCRα subunit gene and 40% amplified the TCRβ subunit gene, while in the B6.NOD*-Aec1/2* mice 66% TCRα and 84% TCRβ were amplified. Of these, TCRα/β paired reads comprised only 12% and 15% for the B6 and the B6.NOD*-Aec1/2* mice, respectively.

**Figure 3:**
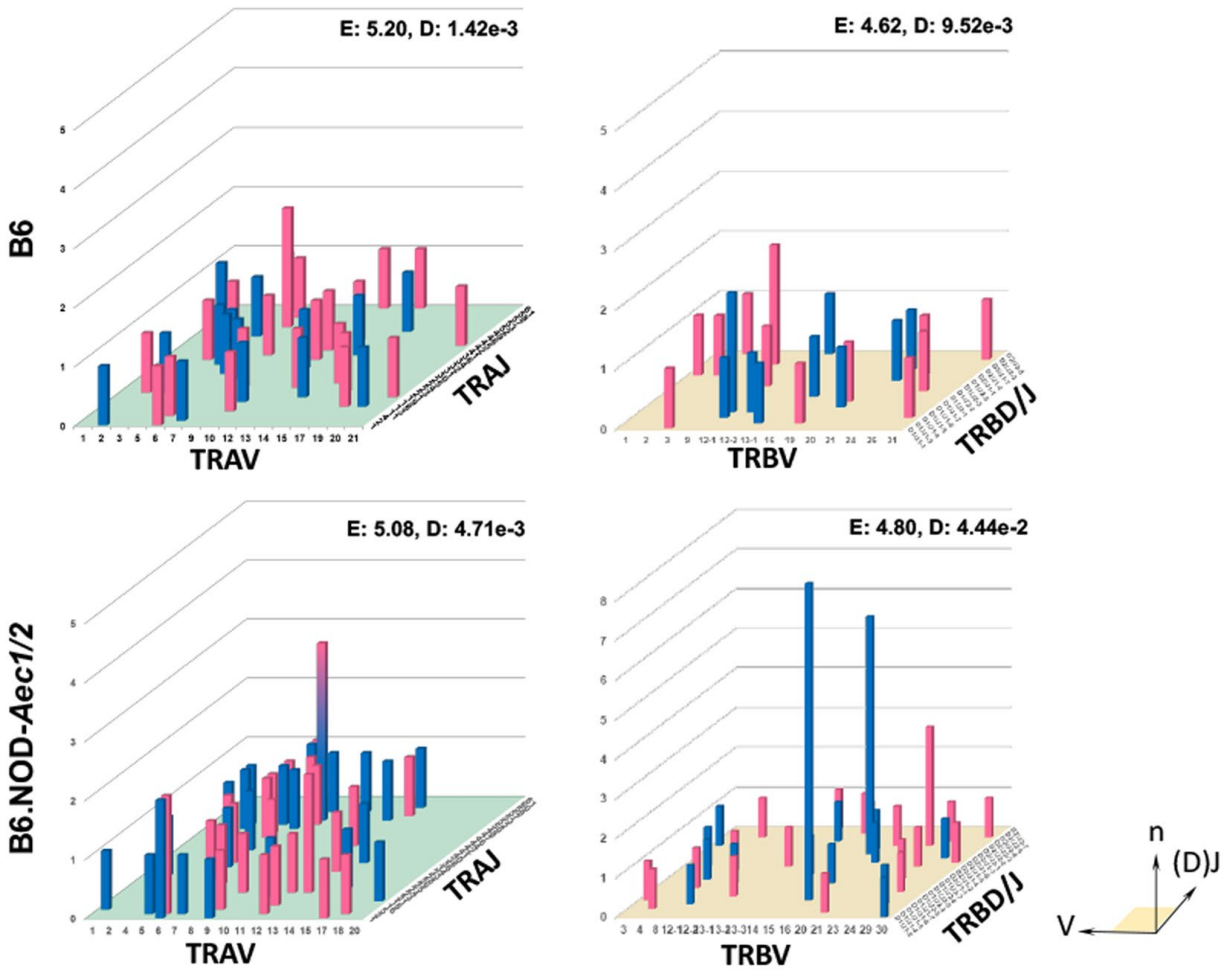
Loss of normal distribution in V/J pairing is associated with the SjS^S^ mouse. Total salivary gland T-lymphocyte three dimensional histograms of TCR gene V/(D)J combinations for B6 TRA (upper left) and B6 TRB (upper right) genes, as well as B6.NOD-Aec1/2 TRA (lower left) and TRB (lower right) genes. Blue bars indicate data from male mice, pink from female mice, and hybrid blue-pink bars for data shared between the sexes. The x-axis represents the analyzed Vα or Vβ gene segment, the z-axis is the analyzed Jα or D/Jβ gene segments, the height or y-axis indicates the total reads of specific V/(D)J combination. V and J genes were designated by the disambiguated IMGT nomenclature for each gene subgroup. TCR repertoire was quantified by entropy (E = Shannon’s Entropy) and diversity (D = Simpson’s Index).

The diversity of the unique V/(D)J combinations in each group’s TCR gene pool reflects the progression of the immune response; a lower diversity indicates clonal expansion and positive selection of antigen experienced, autoimmune cells. Shannon’s Entropy depicts the diversity of a population by measuring uniformity; higher values are more uniform and thus normally distributed. In contrast, the Simpson’s index approximates diversity and indicates overlap, where a lower value indicates greater diversity and 0 indicates no clonal overlap. V/J combinations are catalogued in Fig. 3 as a representation of the repertoire of infiltrating T cells of male and female mice. A complete normal distribution would show a similar frequency of pairings of all of the V/J gene combinations, however it is only likely to see this distribution if all genes are used equally and if thousands of isolated cells are sequenced. B6 mice showed a more heterogeneous distribution of V/J parings across the TRα repertoire compared to B6.NOD*-Aec1/2*, which showed a higher Shannon’s entropy (E: 5.20 vs 5.08) but slightly lower Simpson’s diversity (1.42e-3 vs 4.71e-3). Interestingly, the B6.NOD*-Aec1/2* mice showed a gene bias towards TRAV genes 1–13. There was only one high frequency pairing from the B6 mice, TRAV6/TRAJ52 observed in female mice, whereas the B6.NOD*-Aec1/2* mice displayed pairing from: TRAV8/TRAJ52 (both males and females, indicated by the hybrid pink-blue bar), TRAV6/TRAJ1 (males) or TRAJ2 (females), and TRAV13/TRAJ23 (females). B6 mice showed a narrower distribution of TRβ (E: 4.62 vs 4.80) but had fewer high frequency pairings while B6.NOD*-Aec1/2* mice had two high frequency pairs: TRBV16/(TRBD1/2)TRBJ1-7 from males and TRBV23/(TRBD2)TRBJ2-2 from females with potential TRBV bias toward TRBV genes 3–20 in males, while females showed an opposing bias toward later TRBV genes 12–30. The presence of high frequency V/J pairing in B6.NOD*-Aec1/2* mice in combination with lower entropy, and higher diversity, indicated a restricted TCR repertoire in SjS^S^ mice.

### Distribution V/J pairing contributed by effector Th1, Th17, Tc1 and Tc17 cells

**Figure 4:**
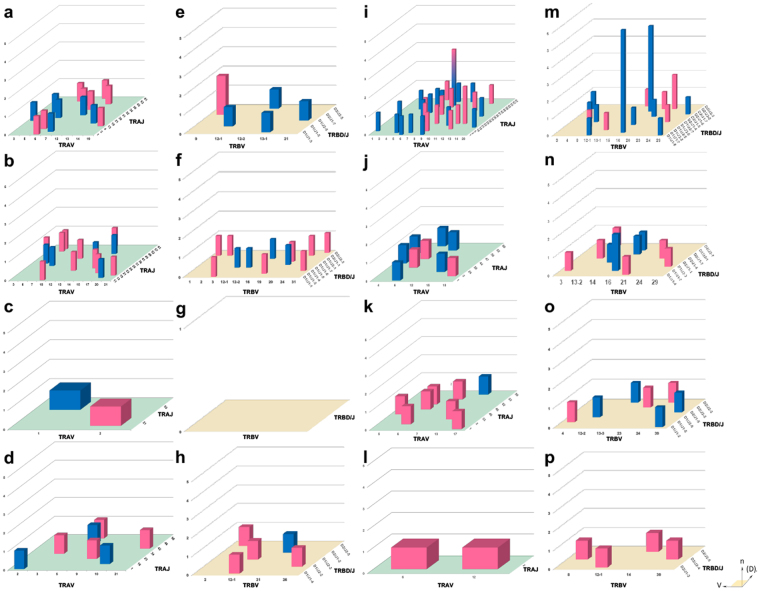
T cells from the SjS^S^ mouse show reduced diversity in V/J pairing. Salivary gland T-lymphocyte three dimensional histograms of TCR gene V/(D)J combinations derived from B6 Th1 cells (**a**) TRA and (**e**) TRB genes, Th17 cells (**b**) TRA and (**f**) TRB genes, Tc1 (**c**) TRA and (**g**) TRB, and Tc17 (**d**) TRA and (**h**) TRB genes as well as B6.NOD-Aec1/2 derived Th1 (**i**) TRA and (**m**) TRB genes, Th17 (**j**) TRA and (**n**) TRB genes, Tc1 (**k**) TRA and (**o**) TRB genes, and Tc17 (**l**) TRA and (**p**) TRB genes. Blue bars indicate data from male mice, pink from female mice, and hybrid blue-pink bars for data shared between the sexes. The x-axis represents the analyzed Vα or Vβ gene segment, the z-axis is the analyzed Jα or D/Jβ gene segments, the height indicates the total reads of specific V/(D)J combination. V and J genes were designated by the unambiguous IMGT nomenclature for each gene subgroup.

Next we looked at the contribution of Th1, Th17, Tc1 and Tc17 cells to the overall repertoires. In Fig. 4, B6 mice have an even distribution of TRAV/TRAJ pairings from Th1 and Th17 cells (Fig. 4a,b) with little contribution from Tc17 cells (Fig. 4d) and even less contribution from Tc1 cells (only 2 TRA pairs and 0 TRB pairs, Fig. 4c,g). The TRAV6/TRAJ52 pairing identified in Fig. 3 was evenly divided between Th1 and Th17 populations. SjS^S^ B6.NOD-*Aec1/2* mice have shown an extensive repertoire of V/J pairings from Th1 cells (Fig. 4I,m) and to a lesser extent Th17 (Fig. 4j,n) and Tc1 cells (Fig. 4k,o); the latter of which had a limited contribution from males. There was no contribution from Tc17 cells (Fig. 4l,p). The TRAV8/TRAJ52 pairing contributed solely to the repertoire of the SjS^S^ B6.NOD-*Aec1/2*, Th1 cells and existed in both female and male mice. The TRAV6/TRAJ1 combination seen previously in Fig. 3 was divided between Th1 and Th17 repertoires of male mice and the TRAV6/TRAJ2 consisted of Tc1 and Tc17 in females. Another pairing unique to the SjS^S^ B6.NOD-*Aec1/2* mice, TRBV16/(TRBD1/2)TRBJ1-7 was present only in the male mice and contributed mostly to the Th1 (with pairings consisting of either TRBD gene) and in a minor extent the Th17 repertoires. The TRBV23/(TRBD2)TRBJ2-2 combination was comprised of Th1 and Tc1 cells derived from female SjS^S^ B6.NOD-*Aec1/2* mice. The SjS^S^ B6.NOD-*Aec1/2* mice display a pattern of expansion of Th1 and Th17 cells from TRAV8/TRAJ52 and TRBV16/(TRBD1/2)TRBJ2-2 pairings in male SjS^S^ mice and Th1 cells from TRAV8/TRAJ52, TRAV13/TRAJ23, and TRBV23/(TRBD2)TRBJ2-2 pairings in female SjS^S^ mice.

### High frequency of CDR3 sequences were only found in T cells of the SjS^S^ B6.NOD-*Aec1/2* mice

Since non-template N nucleotides yield higher diversity, it was utilized to examine the diversity within the CDR3 sequence and hence the TCR repertoires. B6 mice had no identical CDR3α/β clones (Supplementary Tables S1 and S2 **)**. B6 mice had only two premature stop codons (5.9% of CDR3α chain repertoire and 16.7% of the CDRβ chain repertoires) in the IFN-γ producing Th1 cells, whereas SjS^S^ B6.NOD*-Aec1/2* Th1 cells produced 13.2% premature stop codon for the CDR3α chain and 7.4% for the CDR3β chain while Th17 cells produced 20.1% for the CDRα chain and 12.6% for the CDRβ chain repertoire indicative of nonfunctional TCRs (Supplementary Tables S3 and S4). CDR3α and CDR3β of Th1 and Tc1 cells from B6 mice had an unusually low repertoire entropy (4.09 and 2.59, Supplementary Table S1) compared to the SjS^S^ B6.NOD-*Aec1/2* counterparts (5.34, 3.95, Supplementary Table S3) likely due to a smaller B6 repertoire. As expected, Th17 and Tc17 cells of B6 mice had greater entropy (α: 4.59, β: 4.09, Supplementary Table S2) compared to B6.NOD-*Aec1/2* mice (CDR3α: 3.91, CDR3β: 3.88, Supplementary Table S4). There was no overlap in clones in the B6 Th1 and Tc1 sampling (Simpson’s Index values of 0 for both α and β chains) compared to SjS^S^ B6.NOD-*Aec1/2* (0.004, 0.063). Likewise, the Simpson’s Indices indicated no overlap in the CDR3α repertoire and little overlap in the CDR3β repertoire of SjS^S^ Th17 and Tc17 cells (0.000 vs 0.008). In the B6.NOD*-Aec1/2* mice, TRAV8/TRAJ52 (6.7%) and TRAV13/TRAJ23 (4.4%) pairings identified above were translated into CATDLNTGANTGKLTFG and CVYLEHHFE respectively, in which the former clone was derived from non-identical DNA sequences. CDR3β chain translation produced two clones of interest derived from combinations of TRBV16/(TRBD1/2)TRBJ1-7 (CGGKRRLESIFR, 22.2%) and TRBV23/(TRBD2)TRBJ2-2 (CRKLHSCATCALNFL, 7.4%). The former clone (CGGKRRLESIFR) was derived from multiple CDR3β sequences produced by Th1 and Th17 cells. A reduction in entropy with greater Simpson’s Indices, SjS^S^ B6.NOD-*Aec1/2* mice exhibit greater clonal expansion which is consistent with the disease process of SjS and other autoimmune diseases.

### Motif analysis reveals high frequency and sexual dimorphism in the hypervariable CDR3 regions in the SjS^S^ B6.NOD-*Aec1/2* mice

MEME analysis (www.meme-suite.org) was utilized to determine the high-frequency motifs present in the CDR3 regions. Prior research has revealed that hydrophobic amino acids in the center of the CDR3 region have been linked to autoreactivity in T cells^36^. MEME analysis here was also utilized to determine which, if any, of the effector T cell clones of SjS^S^ B6.NOD-*Aec1/2* mice produced TCRs with this specific pattern. The core amino acids LEHH (positions 4–7) in the first motif from Table 2 have a hydrophilic amino acid, glutamate, flanked by leucine (hydrophobic) and two histidines (moderately hydrophobic). Likewise, the core amino acids from the last CDR3 region (SCAT in positions 6–9) contain two moderately hydrophobic amino acids (cysteine and alanine) flanked by two hydrophilic ones (serine and threonine). These two motifs, CVYLEHHFE and CRKLHSCATCALNF, were derived from clones with 100% DNA identity, which indicates that they are a product of clonal expansion. The second motif’s core is NTG (A or T) N in positions 6–10 yielding only one or two hydrophobic amino acids (glycine and alanine), however the glycine in position 8 is conserved among the clones analyzed. The final motif has a central KRRLE motif in positions 4–8, only one of these being hydrophobic (leucine), however this motif contains high confidence in lysine-arginine-arginine positively charge peptides. Three motifs analyzed in B6.NOD-*Aec1/2* mice were present in female Th1 cells, where one (CATDLNTGANTGKLTFG) was also present in males and only one of these (CGGKRRLESIFR) came from a Th17 origin. Two CDR3 amino acid sequences derived from identical DNA sequences (CVYLEHHFE and CRKLHSCATCALNFL) and two derived from non-identical sequences (CATDLNTGANTGKLTFG and CGGKRRLESIFR) provide evidence for clonal expansion and clonal selection, in the SjS B6.NOD-*Aec1/2* mouse autoimmune environment.

**Table 2:**
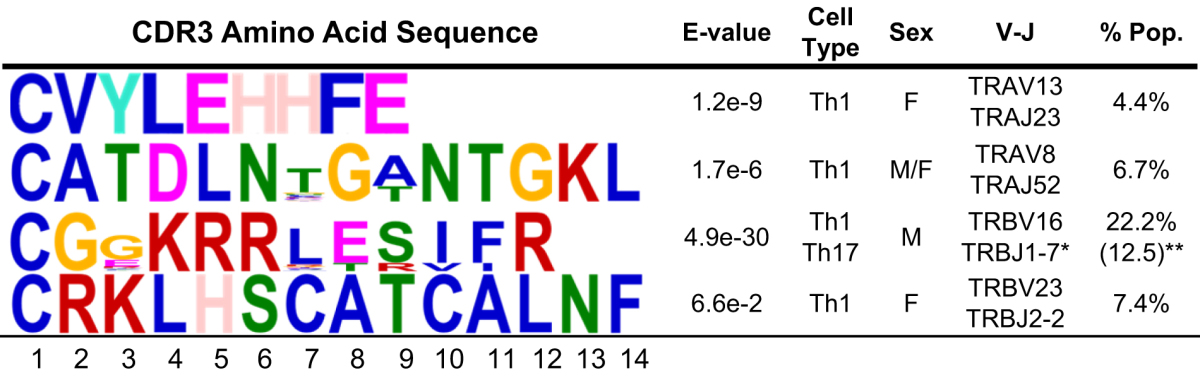
MEME analysis of high frequency CDR3 regions from SjS^S^ mice. Amino acid motif analysis was performed on the high frequency CDR3 sequences from the B6.NOD-*Aec1/2* mice. Bit height corresponds to the likelihood of the amino acid in each position. Blue – hydrophobic, neutral amino acids, Red – positively charge hydrophilic amino acids, Green – Neutral hydrophilic amino acids, Magenta – negatively charged hydrophilic amino acids, Orange-glycine, Teal-Tyrosine, Pink-Histidine (positively charged moderately hydrophobic). E-value indicates the model confidence for the amino acid in that position. Cytokine indicates the secreted molecule(s) associated with the motif, V/J indicates recombination gene segment pairings, and % pop. Indicates the percent of the motif present in the IFN-γ or the IL-17A producing population. *Most popular V/J combination. **Motif located in both IL-17A and IFN-γ producing populations in both TCRα and TCRβ chains.

**Figure 5:**
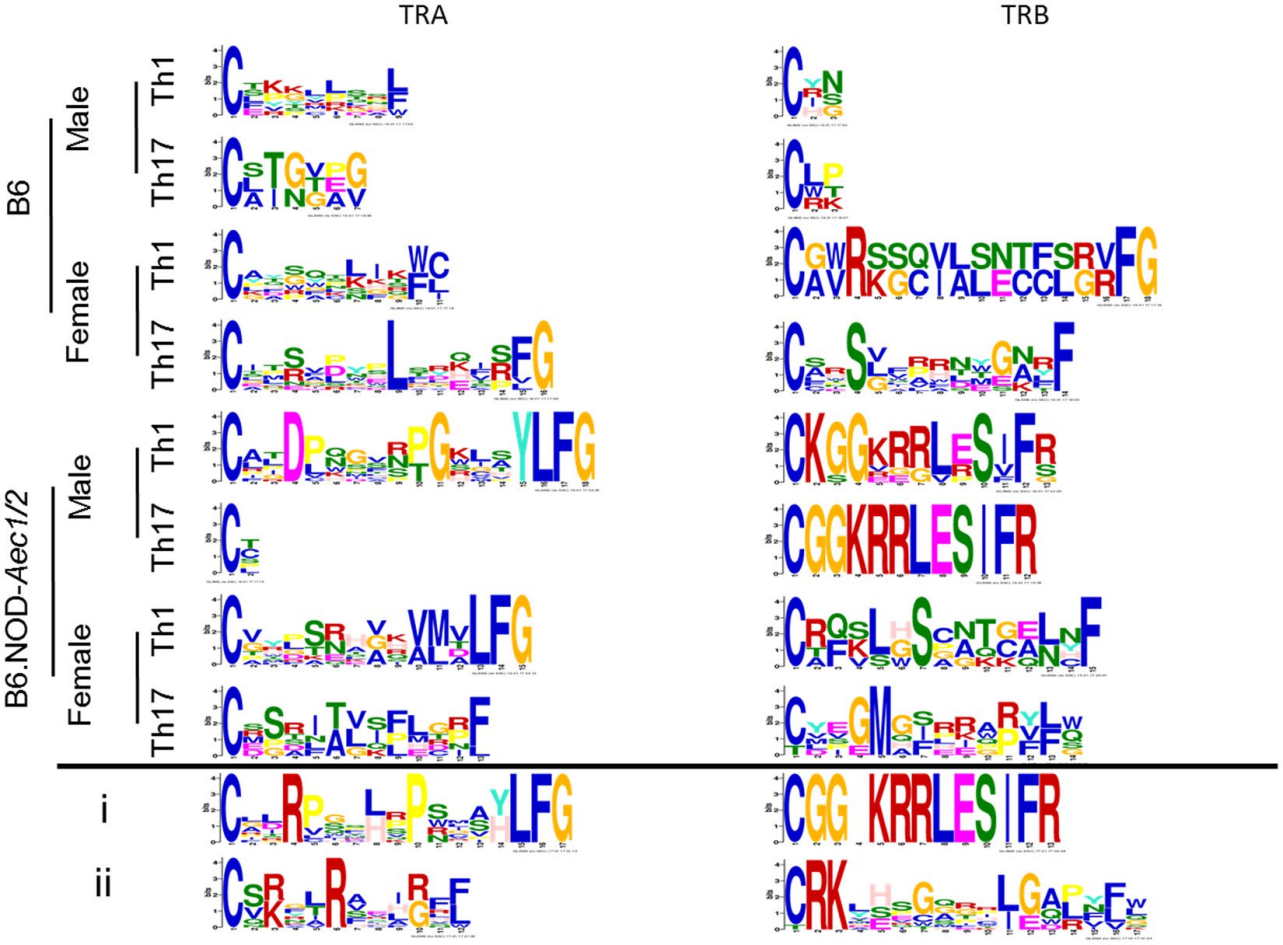
Motif analyses of SjS^S^ mice show conserved amino acids in CDR3 regions of male and female T cells. Glam2 motif analyses were performed on B6 and B6.NOD*-Aec1/2* Th1 and Th17 cell repertoires. Bit height corresponds to amino acid identity likelihood. Blue – hydrophobic, neutral amino acids, Red – positively charge hydrophilic amino acids, Green – Neutral hydrophilic amino acids, Magenta – negatively charged hydrophilic amino acids, Orange-glycine, Teal-Tyrosine, Pink-Histidine (positively charged moderately hydrophobic).

The GLAM2 algorithm (www.meme-suite.org) was employed to determine a full-length motif indicative of each sex and cell type derived from B6 and B6.NOD-*Aec1/2* mice. We analyzed only the Th1 and Th17 repertoires because of the lack of the Tc1 and Tc17 repertoires in B6 mice and the presence of the high frequency CDR3 sequences in Th1 and Th17 cells (Table 2). Repertoires from female B6 mice had only a single leucine conserved in CDR3α peptides from Th1 and Th17 repertoires (Fig. 5), whereas β-chain motifs had an arginine or serine present in Th1 or Th17 repertoires. Several hydrophilic (serine, glutamine, asparagine, or threonine) amino acids are observed in the core of the CDR3α/β chains from B6 mice. Male B6 mice lacked conserved amino acids and produced short MEME analyses. Th1 cells of male SjS^S^ B6.NOD-*Aec1/2* mice contained a motif with an aspartate in the 4th position, either a proline or threonine in the 10th and 11th position of the CDR3α chain motif. Th17 cells from these mice produced abortive MEMEs in this motif analysis. β-chain of both Th1 and Th17 cells from male SjS^S^ B6.NOD-*Aec1/2* mice provided a highly-conserved motif of “CGGKRRLESIFR”. Female B6.NOD-*Aec1/2* mice Th1 cells had either valine, glycine, or alanine in position 8 followed by either a valine or alanine in position 10 and then a methionine or leucine in position 11 of the CDR3α chain, all moderately hydrophobic amino acids. Th17 cells seemed to express serine, proline, or glycine in the 3rd position, threonine or alanine in the 6th position, valine, leucine or glycine in the 7 position and phenylalanine, proline or leucine in the 9 position of the CDR3α chain, again all moderately hydrophobic. Finally, the β-chain of Th1 cells in female SjS^S^ mice showed a motif of leucine or serine in the 5th position and serine in the 7th position, whereas Th17 cells had a glycine or glutamate in the 4th position, methionine in the 5th position, and serine, isoleucine, or phenylalanine in the 7th position. Male B6.NOD-*Aec1/2* mice displayed several cationic and hydrophobic amino acids in the core amino acids of the CDR3α/β chains for Th1 repertoires as well as Th17 CDR3β chain repertoire in contrast to male B6 mice which produced inconclusive motifs. B6.NOD-*Aec1/2* produced an inconclusive motif for Th17 CDR3α chains implying a minimized role of the CDR3α chain selection, however a common motif for both Th1 and Th17 CDR3β chain implies selection. Female B6.NOD-*Aec1/2* mice have a mix of hydrophobic and cationic amino acids (arginine, histidine, valine, and lysine) in the central positions of Th1 CDR3α chain and a mix of polar and nonpolar amino acids in the central positions in contrast with that of the female B6 mice with polar, hydrophilic amino acids. However, both B6 and B6.NOD-*Aec1/2* female mice present similar hydrophilic amino acids (LSNT vs SCNT respectively) in the CDR3β chains of Th1 repertoire. The CDR3α chain of Th17 cells in B6.NOD-*Aec1/2* female mice had motifs of hydrophilic and hydrophobic amino acids in contrast to one conserved leucine in B6 females. Because of contrasting motifs, T cells from male B6.NOD-*Aec1/2* mice are being generated to different auto antigens as that of male B6 mice and female B6.NOD-*Aec1/2* vis-à-vis female B6 mice.

Two representative mice are presented to examine TCR repertoires in a single mouse. The first mouse, a male SjS^S^ mouse (Fig. 5i), had several instances of the “CGGKRRLESIFR” CDR3β sequence (Table 2) as shown by their overall β-chain motif analysis. Motif analysis of the α-chain revealed arginine in the fourth position, leucine or histidine in the eighth position, proline in the tenth position, and either a tyrosine or a histidine in the fourteenth position. These were either positively charged or hydrophobic amino acids. This mouse had multiple clones of the “CATDLNTGANTGKLTFG” (Table 2), however this was obscured in the repertoire and not apparent in the overall CDR3α motif. The second mouse, a female SjS^S^ mouse (Fig. 5ii), had a strong motif of arginine or lysine (cationic) in the 3rd position, arginine in the 6th position, and arginine or glycine in the 10th position while, the β-chain had a motif beginning with arginine-lysine in the 2nd and 3rd position, glycine, cysteine or tryptophan in the 7th position, leucine or threonine in the 11th position and glycine or glutamate in the 12th position. The arginine-lysine beginning of the motif matches the CDR3β sequence “CRKLHSCATCALNFL” (Table 2). Based on motif analyses, both the male and female mice selected for CDR3α/β amino acids that were hydrophobic or cationic in the SjS environment.

## Discussion

The development of SjS is a dynamic and temporal process. Following the perturbation of the salivary glands, the influx of immune cells initially by T cell clusters followed by recruitment of B lymphocytes accelerates the gross pathology of the glands. Effector T cell-produced proinflammatory cytokines, IFN-γ and IL-17A, contribute synergistically to the disease process. T cells recognize autoantigens by their TCR, however, there remains speculation in regards to the clonal selection of these T cells based on their TCR repertoires. We sought to determine the clonal diversity and selection of the TCRs expressed by infiltrating IFN-γ and IL-17A-producing effector T cells, between male and female of SjS^S^ B6.NOD-*Aec1/2* mice. Compared to B6 mice, the SjS^S^ mice show restricted Th1 and Th17 TCR repertoires in both males and females, with more highly limited repertoires in Th17 cells of females. Identified here were two TCR motifs present only in SjS^S^ mice that arise from selection (CATDLNTGANTGKLTFG from Th1 cells and CGGKRRLESIFR in both Th1 and Th17 cells) as well as two TCR motifs that show evidence of expansion in Th1 cells (CVYLEHHFE and CRKLHSCATCALNFL).

IFN-γ induces adhesion molecules in the gland including: vascular cell adhesion molecule-1 (VCAM-1), α4β1 integrin, peripheral node addressing, L-selectin and LFA-1, which allows for the influx of inflammatory cells into salivary glands. Global transcription analyses have indicated that a number of chemokines like CCL5, CXCL16 (an IFN-γ regulated chemokine that attracts NK and memory T cells), CXCL9 and CXCL13 transcripts are up-regulated in the salivary glands of SjS^S^ mice at the time of the disease onset^37^. IFN-γ activity is prominent in SG epithelial cells and biopsies from SjS patients^38^. IL-17A is involved in the proliferation, maturation and recruitment of neutrophils during the initial insult to the glands. Furthermore, IL-17A can function as a B cell helper by inducing a strong proliferative response of B cells and triggering antibody production with class switching and germinal center formation^39,40^. In various related autoimmune disorders, IL-17 can be directly correlated with disease severity^41^. Th17 (not Th1 or Th2) cells are highly prevalent in the SG of SjS^S^ mice and human patients, with a strong sexual dimorphism in the mouse model^20,21^. Here we found a significant increase of Th17 cells in SjS^S^ B6.NOD-*Aec1/2* mice with a higher frequency in females versus males, while Th1, Tc1, and Tc17 cells were also expressed in female SjS^S^ mice. These findings are consistent with research into other autoimmune diseases, including experimental autoimmune encephalitis (EAE), where it was found that females present a more robust immune response with elevated cell mediated responses including increased recruitment of Th1, Th17, Tc1, and Tc17 cells like in the glands of female SjS^S^ mice^42,43^. Exogenous androgen treatment has been shown to reduce IL-12 and IFN-γ levels, lowering disease presentation of EAE^44^, due to the protective effect of androgens in certain genetic backgrounds towards inflammation^19,20,45^. Our data showed a disproportionately high amount of IL-17A produced by female SjS^S^ mice. This upregulation could be attributed to the female sex hormone, 17β-estradiol, which reduces the transition of Th17 cells from the draining lymph node to the target organs as in rheumatoid arthritis^46,47^. SjS may bear sensitivity to estrogen by increasing the activation of Th17 cells via IL-23 causing regulatory dysfunction, allowing migration to the glands, and secretion of proinflammatory cytokines^48^.

The clinical manifestation of SjS in patients is attributed by the hyperactivity of B cells as evidenced by ectopic germinal center (GC) formation, hypergammaglobulinemia, and circulating autoantibodies^49–51^. The GC formation is mediated by the function of follicular helper T (Tfh) cells surrounding activated B cells in the follicles^52^. A study by Maehara *et al*.^53^ has indicated that Th2 and Tfh cells represented by their signature cytokines are positively correlated with increased lymphocytic foci and GC. In the mouse models, IL-4 of Th2 cells plays an important role during the clinical phase, while having little or no effect on the pathology associated with the preclinical disease state^54,55^. The mice examined in the study were at the advanced age, mimicking the clinical phase of human SjS which is predominated by Th2/Tfh response. The study did not examine the effector Th2/Tfh cells, but it could explain the low numbers of other effector T cells in the SG. One potential question which has not well understood or investigated in the SG is differentiation of recirculating memory T cells present in the vascular system of the SG and resident memory T cells in the parenchyma. As alluded to earlier, high numbers of effector T cells were identified in the glands of SjS^S^ mice. As presented in Supplementary Fig. S2, the majority of the effector T cells are resident or gland-infiltrating T cells and not from circulation. Further work is needed to address the gland-specific pressure exerted on the enrichment for effector T cells and whether their expansion is initiated from the circulation or secondary lymphoid tissues.

The influx of inflammatory cells into the salivary glands raises concerns regarding the migratory nature as well as the selection process of infiltrating lymphocytes. The danger signals in the epithelium in conjunction with the activation of adhesion molecules and homing chemokines/chemokine receptors drive the flow of immune cells into the glands. There are two scenarios which could explain the migratory pattern of these infiltrating T cells and resulting TCR repertoires. First, there is a massive influx of various effector cell types with polyclonal TCR repertoires. The gland milieu and unique antigen presentation select for monoclonal TCR repertoires expressed on a specific subset of effector T cells, i.e. a selection for only SG antigen-specific T cells with a bias against T cells specific for other antigens. Alternatively, the initial infiltrates are composed of a few sporadic T cells with limited TCR repertoire. These early T cells proliferate and clonally expand, which maintains their monoclonal repertoires. There is no human data that examines the temporal influx of T cells into the SG, however using the SjS^S^ mice, we have observed there is a sporadic infiltration of T cells in younger mice, followed by a massive occupation of T cell during the onset of the disease. This observation, in addition to studies that show reduced TCR diversity in pathogenic T cells in SjS and other autoimmune diseases^56–59^ (which even correlate disease severity with T cell expansion^60^) all appear to support the latter scenario. In some cases, restriction of the TCR diversity is necessary for rapid IL-17 secretion^61^. In the study, SjS^S^ mice exhibited less normal distribution but a higher diversity in the CDR regions. Sequence analysis of the CDRs pointed to the presence of premature stop codons which were mapped to non-templated bases in the CDR regions, therefore they are more likely to represent real events. SjS^S^ mice appeared to show an increase of the premature stop codons in TCRα and TCRβ chains by Th17 cells. As discussed, glandular Th17 cells are antigen-specific and hyperproliferative upon antigen-specific stimulation^62,63^. This finding suggests that hyperproliferation of Th17 generates more error-prone DNA rearrangements, which could generate more premature stop codons. It is possible that nonsense-mediated decay (NMD) that degrades aberrant transcripts harboring premature stop codons are not efficient or defective in SjS^64^, therefore truncated or pathogenic proteins from non-productively rearranged genes are being produced and possibly initiate glandular dysfunction.

The interaction of CDR3 with the SjS autoantigen has yet to be elucidated. However, hydrophobic amino acids in the N regions play a part in binding to autoantigens^36,65^. It is hypothesized that these hydrophobic amino acids promote additional interaction with the MHC-peptide complex to enforce a strong binding. In this manner, our data is consistent, in that hydrophobic and partially hydrophobic amino acids in these positions of the clonotypes were identified in this study; this supports that these CDR3 clonotypes were generated to an autoantigen. Although TCR repertoire analysis is being performed in other disease studies, is limited to CD4^+^ Th or CD8^+^ Tc cells and not effector subsets^56,66^ SjS^S^ mice had a gene bias towards the selection of TRAV1-TRAV13. Though the significance of this bias is uncertain in SjS, the expression of the Th17 transcription regulator, RORγT, is associated with the development and survival of CD4/CD8 double positive thymocytes. RORγT skews TCRα selection towards distal Vα genes (TRAV1-TRAV6) in EAE^67^. Previous studies have identified certain V genes associated with SjS in the SG from humans. Sumida *et al*. identified the biased utilization of TRAV17-1, TRAV2, and TRAV11-1 quoting the use of GGPKT and VDxG motifs as well as TRBV2 and TRBV13 with the STxTLRNEQ motif. Motifs and pairings were unique to each SjS patient. Another study which was done on the NOD mouse concluded that 15% of their TCR repertoire was of TRBV8.1 and TRBV2^68^. Another SjS patient study examining TCRβ observed that seven TCRβ genes were devoid in their set, two of which (TRBV16, and TRBV23) were enriched in our datasets^69^. The most prevalent CDR3 clonotype observed (CGGKRRLESIFR) was present in Th17 and Th1 cells while remaining exclusive to the SjS^S^ male mice. This implies that both Th1 and Th17 cells may have a common selective pressure for autoantigen in the glands. With the divergence of results from the literature, we can see that the relationship between TCR diversity and SjS is still very complex.

This study is the first of its kind to identify sex differences in functionally distinct T cells of autoimmunity. We observed that males develop fewer Th1 and Th17 cells in the glands with unique TCR repertoires from female SjS^S^ mice. Male Th1 and Th17 repertoires select for similar CDR3β amino acids with little selective pressure for Th17 CDR3α while female SjS^S^ mice presented an alternative selection for hydrophobic and cationic amino acids. Furthermore, we have identified four CDR3 motifs which represent motifs associated with the SjS^S^ mice. It is still unclear what causes the sexual bias in SjS, so it is necessary to interrogate the mechanism from which different sexes pose different degrees of selection. The temporal role of sex hormones, the balance of androgen and estrogen in different stages of life, may play a role in disease development and progression, and evident from this study, T cell selection. Lastly, the implications of the specific clonotypes and whether they inherently produce SjS or are simply indicative of the disease is still not understood.

## Methods

### Animals

SjS^S^ C57BL/6 J.NOD/ShiLtJ-*Aec1Aec2* (B6.NOD-*Aec1/2*) and non-SjS^S^ C57BL/6 J (B6) control mice were bred and maintained under specific pathogen-free conditions in the animal facility of Animal Care Services at the University of Florida. The breeding and the use of animals described herein were approved by and carried out within accordance of the University of Florida’s Institutional Animal Care and Use Committee. All methods were performed in accordance with the relevant guidelines and regulations. Development of the B6.NOD-*Aec1/2* mouse and its SjS-like disease phenotype are described elsewhere^70,71^. All animals were maintained on a 12-hour light-dark schedule and provided food and acidified water *ad libitum*. At times indicated in the study, mice were euthanized by cervical dislocation after deep anesthetization with isoflurane and their organs and tissues freshly harvested for analyses.

### Single-cell microengraving process

Salivary gland lymphocytes of B6 and B6-*NOD*.*Aec1/2* mice were isolated as previously described^19^. Lymphocytes were cultured in RPMI complete medium (12-167 F, Lonza) containing 10% fetal bovine serum (F4135, Sigma-Aldrich), 100 U/mL penicillin, 100 μg/mL streptomycin (SV30010, GE Life Science), 2 mM L-glutamine (25-005-Cl, Corning), 10 mM HEPES (SH30237.01, GE Life Science), 1X MEM-non essential amino acids (25-025-Cl, Corning), 1 mM pyruvate (SH30239.01,GE Life Science), and 0.0004% 2-mercaptoethanol (M3148, Sigma-Aldrich), under T-cell stimulation: 5 ug/mL anti-CD28 (553294, BD Biosciences) and 10 ug/mL anti-CD3 (145-2C11, BD Biosciences) for 64 hours at 37 °C. Following stimulation, lymphocytes were treated with 500 ng/mL Ionomycin sulfate (I3909-1ML, Sigma-Aldrich) and 50 ng/mL phorbol 12-myristate 13-acetate (PMA: P1585-1MG, Sigma-Aldrich), blocked using anti-CD16/32 TruStain FcX blocker (101320, Biolegend) for 5 minutes on ice, and then stained with cell surface markers, anti-CD3-Alexa Fluor 647 (HM3421, Invitrogen), anti-CD4-fluorescein isothiocyanate (FITC) (MCD0401, Invitrogen), anti-CD8-phycoerythrin (PE) (MCD0804, Invitrogen) as well as Calcein violet 405 nm Live/Dead marker (C4858,Invitrogen) for 30 minutes. Following surface staining, 500,000 cells were suspended in 300 μL complete medium were deposited onto the arrays of nanowells and imaged as previously described^72^. The nanowells were hybridized with the capture slide containing antibodies against IL-17A (AAM60, AbD Serotec) and IFN-γ (505827, Biolegend). After 2 hours, the capture slide was treated with anti-IL-17A conjugated with Dylight 594 (46413, Thermo Scientific) and anti-IFN-γ conjugated with Dylight 488 (46403, Thermo Scientific). The slide was imaged using the Genepix 4400 A scanner (Molecular Devices). Single-cell data analysis was performed as described previously^72^.

### Sequencing of TCRs using nested polymerase chain reaction (PCR)

Cells expressing either CD4 or CD8 surface markers and either IFN-γ or IL-17A cytokines were collected using a Narashige micromanipulator loaded with a pulled glass capillary. Single, isolated cells were pipetted into PBS with 2% BSA and 200 U/mL Rnasin RNAse inhibitor (N251A, Promega) and frozen at −20 °C. Frozen single cells were directly used for reverse transcription (RT) reaction per manufacturer’s protocol (1708840, iScript cDNA Sythesis Kit, Biorad) supplemented with 0.1% Triton × 100 detergent to achieve cell lysis. Serial dilutions of about 5 cells per reaction were isolated from the spleen following a CD4^+^ enrichment via CD4^+^ untouched cell isolation kit (11141, Miltenyi Biotec) and used as positive controls. Following RT, PCR was performed using a protocol and primers described previously^73^. In short a 1:50 dilution of the cDNA produced from single-cell RT reaction was used as template for PCR using Primestar Taq (R010A, Clontech). Successful reactions were determined by gel electrophoresis and bands size confirmation.

### Bioinformatic analysis of TRA and TRB Hypervariable Region and Motif analyses

Sanger DNA Sequencing was performed by Eton Bio (Raleigh, NC). T receptor gene and allele identification was determined using the IgBLAST tool (http://www.ncbi.nlm.nih.gov/igblast/). CDR3 sequences were determined using an in-house macro developed for use with Microsoft Excel. The macro was assembled to designate the sequence between the codon for cysteine (TGY) followed by the DNA sequence TTYGGX (where Y denotes either pyrimidine and X denotes any nucleic acid) as the CDR3 sequence. Only sequences less than 60 nucleotides were considered. This allowed for a level of stringency to identify CDR3 regions of high diversity. In some cases, unambiguous germline genes could not be defined due to insertions and deletions, however those sequences containing a canonical CDR3 sequence were retained for analysis. Motif analysis was performed using www.meme-suite.org website. Analyses of high frequency CDR3 amino acid sequences were performed using the MEME tool (http://meme-suite.org/tools/meme) to identify conserved amino acid motifs while the GLAM2 tool (http://meme-suite.org/tools/glam2) was used to identify gapped motifs among diverse datasets.

### Statistical Analysis

Data was analyzed using a Kruskall-Wallis test (one-way ANOVA) with Dunn’s post hoc test. (GraphPad Prism, GraphPad Software Inc.) to determine to determine the statistical significance between female B6.NOD-*Aec1/2* mice from each of the other groups. Significance was defined as a *p-value < 0.05, **p < 0.05, and ***p < 0.005. TCR Clonal diversity was determined with Shannon’s entropy as well as Simpson’s Diversity Index.

## Electronic supplementary material

Supplementary Tables and Figures
