# Supplementary material for: Single-cell analysis reveals sexually dimorphic repertoires of Interferon-γ and IL-17A producing T cells in salivary glands of Sjögren’s syndrome mice
Source: Sci Rep. 2017 Oct 2;7:12512. doi: 10.1038/s41598-017-12627-6 (PMC5624952; doi:10.1038/s41598-017-12627-6)
Supplement: Supplementary file 1 — Supplementary Tables and Figures [file 41598_2017_12627_MOESM1_ESM.pdf]

Single-cell analysis reveals sexually dimorphic repertoires of Interferon-γ and IL-17A producing T cells in salivary glands of Sjögren's syndrome mice

Arun Wanchoo[1], Alexandria Voigt[1], Sukesh Sukumaran[2], Carol M. Stewart[3,5], Indraneel Bhattacharya[3,5], *Cuong Q. Nguyen[1,4,5]

[1]Department of Infectious Diseases and Pathology, College of Veterinary Medicine, University of Florida, Gainesville Florida, USA, [2]Rheumatology Section, University of Arkansas for Medical Sciences, Arkansas Children's Hospital, Little Rock Arkansas, USA, [3]Department of Oral and Maxillofacial Diagnostic Sciences, [4]Department of Oral Biology, [5]Center of Orphaned Autoimmune Diseases, University of Florida, Gainesville Florida, USA

Table 1. Hypervariable regions of IFN-γ expressing T cells from B6 mice

Sex	Age (wk)	T cell	TRAV	CDR3α	TRAJ	%	TRBV	CDR3β	TRBD	TRBJ	%
M	32	Th1	6-1	CKGMRGGFRIFPFGKKS	30	5.9	12-2	CISENASAEGGP		2-5	16.7
F	56	Th1	7N-4	CGHRYTLGRCSGMFL	36	5.9	9	CGWRKSCVASECFLGRFG		1-7	16.7
F	56	Th1					4	CLCFC	1		16.7
M	60	Th1					13-1	CRNILRGSREVK	1		16.7
M	60	Th1					12-1	CCYNVDFLPFS			16.7
M	60	Th1					21	CHGWS*GRRHLANKRATHLAPFG		2-5	16.7
M	51	Tc1	1	CTKKVIQHLRDFG	53	5.9					
F	56	Tc1	2	CPTSFLLHSLYPIFS	21	5.9					
F	60	Th1	3D-3	CKDPQPNIKWCLGNPL	21	5.9					
M	60	Th1	4-4	CRSRLSNLRHRLRF		5.9					
F	60	Th1	5-1	CLYSANKKGCF	32	5.9					
F	60	Th1	6-4	CAAWWKKIQCIRVIFRPSGVW	1	5.9					
M	60	Th1	6D-6	CEPGLPTALIRKLFC	52	5.9					
F	56	Th1	7	CSLT*PPSLS*LGK	2	5.9					
M	32	Th1	12-3	CSVTHKDSLVVSGNPSF	31	5.9					
F	60	Th1	12D-2	CAQGGCLITL	34	5.9					
F	60	Th1	12D-3	CMRYCEPFL	61	5.9					
M	60	Th1	13-3	CLPPLLLPWLSGVFRLF	45	5.9					
M	60	Th1	14-2	CFYYCLSDFS	19	5.9					
M	32	Th1	19	CKKYPKRF	13	5.9					
F	56	Th1	1	CAVRSGQILLNTCSRVFG	25	5.9					
**Shannon's Entropy:**				**4.09**				**2.59**			
**Simpson's Index:**				**0.000**				**0.000**			

Listed are translated CDR3 amino acid sequences from IFN-γ producing T cells isolated from the B6 mouse indicating the Sex, Age (in weeks), T cell (Th indicates CD4 expression, Tc indicates CD8 expression), TRAV (unambiguous IMGT gene name of the TRAV), TRAJ, TRBV, TRBJ and the % (percent of the repertoire) of the individual instance of each clone. Shannon's Entropies and Simpson's indices indicated are calculated from the repertoire of the CDR3α and the CDR3β separately.

Table 2. Hypervariable regions of IL-17A expressing T cells from B6 mice

Sex	Age (wk)	T cell	TRAV	CDR3α	TRAJ	%	TRBV	CDR3β	TRBD	TRBJ	%
F	56	Th17	12D-2	CTSVDYLRILLSHLSFA	41	4.2	19	CPSVPALGKR		1-3	5.9
F	56	Th17	14D-3/DV8	CLVNSSLQFFR	61	4.2	2	CSRSVRRDEGRAGL		1-1	5.9
F	60	Th17	20	CWAELPTRPRGNSPAFG	13	4.2	3	CTSWRHSWGYF		1-1	5.9
F	56	Tc17	21/DV12	CAPSQRNPLRSRFL	42	4.2	3	CIKEEIDFQ			5.9
F	60	Th17		CGVWQFDSFW		4.2	1	CAHVFVRDGNTFG	2	1-1	5.9
F	56	Tc17					2	CNFHNGARFELKCFRFG	2	2-5	5.9
M	32	Th17					12-1	CLPIYLFFV	1		5.9
F	56	Tc17					12-1	CLRIPLTSYFA	1		5.9
M	51	Th17					16	CLPSTVGIAPLPNRGLAIRFG		2-1	5.9
F	60	Th17					17	CFHSGFAFNYGNFFR		1-3	5.9
M	51	Th17					20	CGHVRACWKAFN	1	1-6	5.9
F	60	Th17					20	CISAAWNMEALFG		1-7	5.9
M	60	Tc17					21	CRTSTLGTLRHHLRLGVLFG		1-2	5.9
F	60	Th17					24	CCCESLLHLFF			5.9
F	56	Tc17					26	CMIFTFIFYYSIHFP		2-2	5.9
F	56	Th17					31	CAWSLHRRNER	1	1-4	5.9
F	60	Th17					31	CSRSQPRTTGLFV		2-3	5.9
M	32	Th17	6D-6	CAIGTEGRSGYF	31	4.2					
F	60	Th17	13D-2	CETSFKPFYKFSFG	23	4.2					
F	60	Th17	15D-2/DV6D-2	CGMADCHTYHLFG	24	4.2					
F	56	Tc17	3-4	CINEKDQWLPGVRFG	34	4.2					
F	60	Th17	14D-3/DV8	CLAYSSTFSFP	36	4.2					
F	60	Th17	6D-4	CLMRASTPARVFN	57	4.2					
M	51	Th17	15D-2/DV6D-2	CLTGVAVKGFG	49	4.2					
F	56	Tc17	10	CMGPNHHVFS	18	4.2					
F	60	Th17	4N-4	CPFPWFGIFG		4.2					
F	60	Tc17	2	CPRDLTESYIVHCGLR	1	4.2					
F	60	Th17	7-5	CQEMERPNRGVSGRGGNF	25	4.2					
F	60	Th17	1	CRFK		4.2					
F	56	Tc17	5-1	CRRFGSATFG	58	4.2					
M	60	Tc17	5	CSLLWR-VVFP	46	4.2					
M	51	Th17	3-3	CSTNGPGWQVFR	45	4.2					
F	60	Tc17	9N-3	CSYVEERDAPFG	23	4.2					
F	56	Th17	10	CTSGYTDFV	11	4.2					
F	60	Th17	6-2	CTTNILYDLKRETFG	52	4.2					
M	60	Th17	12D-2	-		4.2					
**Shannon's Entropy:**				**4.59**				**4.09**			
**Simpson's Index:**				**0.000**				**0.000**			

Listed are translated CDR3 amino acid sequences from IL-17 producing T cells isolated from the B6 mouse

indicating the Sex, Age (in weeks), T cell (Th indicates CD4 expression, Tc indicates CD8 expression), TRAV (unambiguous IMGT gene name of the TRAV), TRAJ, TRBV, TRBJ and the % (percent of the repertoire) of each individual instance of that clone. Shannon's Entropies and Simpson's indices indicated are calculated from the repertoire of the CDR3α and the CDR3β separately.

Table 3. Hypervariable regions of IFN-γ expressing T cells from B6.NOD-*Aec1/2* mice

Sex	Age (wk)	T cell	TRAV	CDR3α	TRAJ	%	TRBV	CDR3β	TRBD	TRBJ	%
M	64	Th1	6-4	CIGPSFPISFKGPTHVQYL	34	2.2	16	CGGKRRLESIFR‡	2	1-7	22.2
F	20	Th1	8D-1	CATDLNTGANTGKLTFG†	52	6.7	13-1	CASSGGGTGQLYFG	1	2-2	3.7
F	20	Tc1	5-1	CLLLVFLFFFFFS	40	2.2	6	CLFKEDNPSPF			3.7
F	20	Tc1	6-4	CPHPRPSRLRVVLFFS	46	2.2	4	CGPGQGWLGDPIVLRAF			3.7
F	20	Th1	6N-7	CENNTHYIRLDTVFS	36	2.2	7-5	CGGGG**RIFKILEFG	2	1-1	3.7
M	60	Th1	7-4	CPPPKLGVSLHLF*V*QPSPFL		2.2	20	CDLW	2	1-5	3.7
F	20	Th1	13D-1	CRFSQKKLFIIGFLLGI*DEFG	53	2.2	21	CYRTSPHLFW		1-4	3.7
F	20	Th1					23	CRKLHSCATCALNFL		2-2	7.4
M	64	Tc1					2-1	CKGGVGVPSFFG			3.7
M	64	Th1					3	CINIR		2-2	3.7
M	64	Tc1					3	CPFG		2-6	3.7
M	60	Th1					4	CAHDWDSNHVHIVW		1-4	3.7
M	60	Th1					8	CAHGLGVPINVHIVW	1	1-4	3.7
F	60	Th1					8	CTFVLGSPNQGENCFG			3.7
M	64	Th1					12-1	CTPLFQ		1-6	3.7
M	64	Tc1					12-2	CRSKSNPLLSYITLFP	1	2-6	3.7
M	64	Tc1					13-3	CEALLSGEERLRVFS			3.7
F	38	Th1					20	CRQSLWANKKENHFF*KLW	2	2-2	3.7
F	20	Tc1					24	CLGGIILFLW	2		3.7
M	64	Th1					29	CRYQHI*FC		1-6	3.7
F	20	Tc1					30	CCLDRFF			3.7
F	20	Th1	13D-2	CVYLEHHFE	23	4.4					
M	64	Th1	1	CGGKRRIESISESMGFD	5	2.2					
M	64	Th1	2	CALVWDSNHVHLFG		2.2					
M	64	Th1	2	CPDCGCRISFG	28	2.2					
M	64	Th1	4	CGPNKGDDWIR	48	2.2					
M	64	Th117	4N-4	CFRPQPHRPWCAFG	44	2.2					
M	64	Th1	5	CSPLPPPVAGQGRSL	2	2.2					
F	60	Th1	8	CLGSNHGQLFG	38	2.2					
F	60	Th1	8	CRDILLMVPIM*DCTRS	50	2.2					
M	64	Th1	11	CAIPIFLVFLG	21	2.2					
F	38	Th1	16	CSSGGGVVCGEPFG		2.2					
F	38	Tc1	17	CPLPALV*ILL	1	2.2					
M	64	Th1	20	CLCLLPGSCAFL	18	2.2					
M	60	Th1	6-4	CPPR*CRF	50	2.2					
F	20	Tc1	6-5	CSFTEIVW	52	2.2					
F	60	Th1	6-5	CTWLESNHVQLFG	61	2.2					
M	60	Th1	6-7	CKDKDGLAQEVMK	41	2.2					

F	20	Tc1	6N-5	CSCLSLALLLPAPLG	2-1	2.2
M	64	Th1	7-3	CRLPHMLPPSGPFG	48	2.2
F	20	Th1	7-3	CYPVGTRASFS	25	2.2
F	60	Tc1	7D-6	CGHDPHTP*TRAAIVMVLFW	53	2.2
M	60	Th1	7D-6	CMTYLRVKGTSGL	2	2.2
M	64	Th1	8-2	CFFVFG	57	2.2
F	20	Th1	9D-2	CVMGVFQGVSRHRLFG	5	2.2
F	60	Th1	9D-4	CSRSNEVKMDF	23	2.2
M	64	Th1	9N-3	CGDPQNVGALFG	1	2.2
M	64	Th1	10D	CLILSSRRMSYLFI	57	2.2
F	20	Th1	12-1	CFVFKENSG	42	2.2
F	60	Th1	12-2	CSGQNPLEN*TFL	16	2.2
F	60	Th1	12-3	CWLWQVSRTASPEK	23	2.2
M	64	Tc1	12-3	CQAEEQGTFR		2.2
M	64	Tc1	13D-2	CTPGVSPLSFR	59	2.2
F	38	Tc1	13N-3	CTVRIFALMIW	33	2.2
M	66	Th1	14D-3	CPDSNTF	36	2.2
F	60	Th1	15D-1	CGRPKRQAHAALF		2.2

**Shannon's Entropy:**	**5.34**	**3.95**
**Simpson's Index:**	**0.004**	**0.063**

Listed are translated CDR3 amino acid sequences from IFN-γ producing T cells isolated from the B6.NOD-*Aec1/2* mice indicating the Sex, Age (in weeks), T cell (Th indicates CD4 expression, Tc indicates CD8 expression), TRAV (unambiguous IMGT gene name of the TRAV), TRAJ, TRBV, TRBJ and the % (percent of the repertoire) of each individual instance of that clone. Shannon's Entropies and Simpson's indices indicated are calculated from the repertoire of the CDR3α and the CDR3β separately. †Indicates a CDR3 sequence generated from noncongruent DNA sequences. ‡Indicates a CDR3 sequence generated from multiple V/J gene segment combinations and noncongruent DNA sequences.

Table 4. Hypervariable regions of IL-17A expressing T cells from *B6.NOD-Aec1/2* mice

Sex	Age (wk)	T cell	TRAV	CDR3α	TRAJ	%	TRBV	CDR3β	TRBD	TRBJ	%
M	64	Th17	6	CLVGTQSRALFG	1	6.7	16	CGGKRRLESIFR‡	2	1-7	12.5
F	20	Tc17	16	CFIFLFC		6.7	5	CYEEGSERGPFFG			6.3
F	20	Th17	18	CESRNTVSHRNFERTLHFG	2	6.7	6-3	CDIGAFHIWPFFQGGLFR		1-4	6.3
F	38	Th17	6-4	CSSTLTLKLMCIFFFG	26	6.7	15	CQQTEKGGAQNGGRPG		2-3	6.3
F	20	Tc17	12-2	C*SAIAGIFLGPFG	2	6.7	23	CTQWQRPPNVKGVFL	2	2-2	6.3
F	20	Th17		CTSGAACLFL		6.7	24	CVPGQRRARVLSRRFL		2-7	6.3
F	20	Th17					2	CCLVFFFFC			6.3
F	20	Th17					3	CYSGGPPRVLQKGREKKEGIFL		1-7	6.3
F	38	Tc17					8	CT*LEPKKSAMFW	2	2-4	6.3
M	64	Th17					14	CHYPELVC*FW		1-1	6.3
F	20	Th17					24	CFLCPFG		1-4	6.3
F	20	Th17					29	CEVPGFL		2-3	6.3
F	20	Tc17					30	CMEGMGILEARYLWG		2-4	6.3
F	20	Tc17					12-1	CSIAATLVGHRKQPYFW	2		6.3
F	20	Th17					13-2	CSYRTTYVFG	2	2-7	6.3
M	64	Th17	4-3	CCGDRPSKKMIF	50	6.7					
M	60	Th17	7	CCQRA*SFL	32	6.7					
M	64	Th17	6N-7	CPSLKLAHLVKPQYFL	60	6.7					
F	20	Tc17	6N-7	CDSRIAVIFLDRLAHL	2	6.7					
F	20	Th17	6-7/DV9	CMGSFAQLGRFG	47	6.7					
M	52	Th17	12-3	CTHDSLFG	52	6.7					
M	52	Th17	15-1/DV6-1	CNPVYGPGSCTFW	24	6.7					
F	20	Tc17		CRPDITLSPETPFL		6.7					
M	64	Th17		CSALPRG*PLW		6.7					
**Shannon's Entropy:**				**3.91**				**3.88**			
**Simpson's Index:**				**0.000**				**0.008**			

Listed are translated CDR3 amino acid sequences from IL-17A producing T cells isolated from the B6.NOD-*Aec1/2* mice indicating the Sex, Age (in weeks), T cell (Th indicates CD4 expression, Tc indicates CD8 expression), TRAV (unambiguous IMGT gene name of the TRAV), TRAJ, TRBV, TRBJ and the % (percent of the repertoire) of each individual instance of that clone. Shannon's Entropies and Simpson's indices indicated are calculated from the repertoire of the CDR3α and the CDR3β separately. ‡Indicates a CDR3 sequence generated from multiple V/J gene segment combinations and noncongruent DNA sequences.

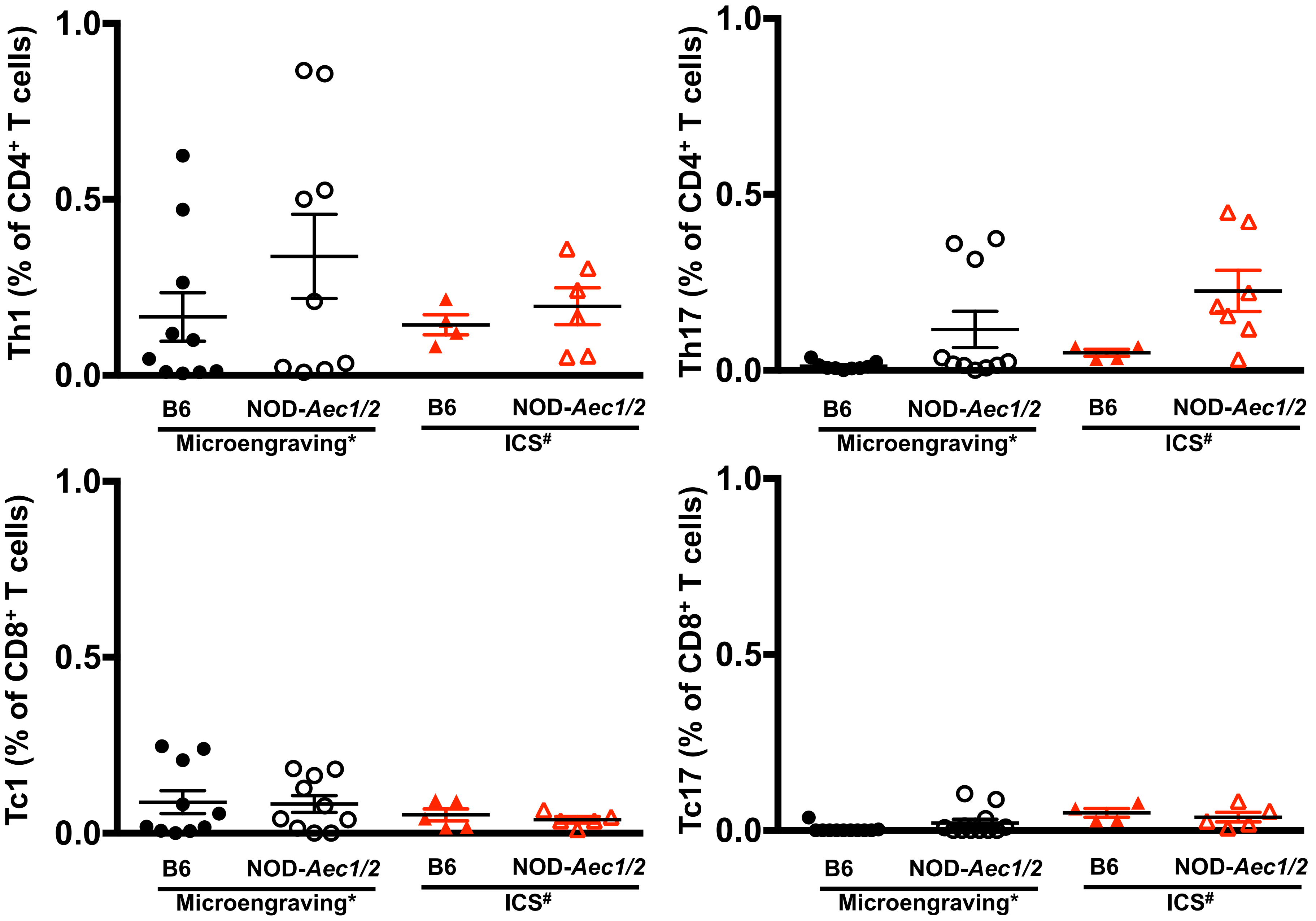

Th1 (% of CD4+ T cells)
1.0
0.5
0.0
B6
NOD-Aec1/2
Microengraving*
ICS#
Th17 (% of CD4+ T cells)
Tc1 (% of CD8+ T cells)
Tc17 (% of CD8+ T cells)

Supplementary Fig. S1. Similar frequency/trend of cytokine secretion by microengraving and direct *ex-vivo* ICS. Microengraving was performed as stated in Methods section. The frequency in percentage of microengraving was determined by using the percentage (multiplied by 100) of the total number of Th1 or Th17 cells from wells with single live cells among the total number of wells with single $CD4^+$ or $CD8^+$ cells. #Direct *ex-vivo* ICS involves stimulation of salivary gland lymphocytes for 4 hour under PMA/Iono stimulation in the presence of GolgiPlug containing Brefeldin A solution (BD Biosciences). Flow cytometry was used to analyze for CD3, CD4, CD8, IL-17A, and IFN- γ secretion. The frequency in percentage was determined by using the percentage (multiplied by 100) of the total number of Th1 or Th17 cells from wells with single live cells among the total number of wells with single $CD4^+$ or $CD8^+$ cells.

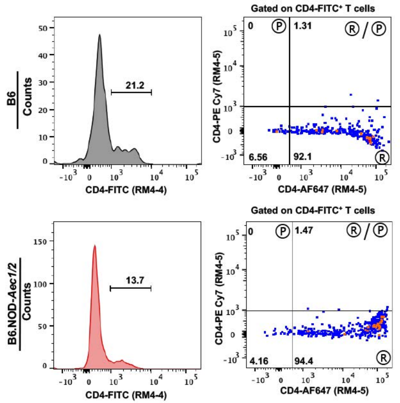

B6
Counts
21.2
CD4-FITC (RM4-4)
Gated on CD4-FITC+ T cells
P
R / P
0
1.31
6.56
92.1
R
CD4-PE Cy7 (RM4-5)
CD4-AF647 (RM4-5)
B6.NOD-Aec1/2
Counts
13.7
CD4-FITC (RM4-4)
Gated on CD4-FITC+ T cells
P
R / P
0
1.47
4.16
94.4
R
CD4-PE Cy7 (RM4-5)
CD4-AF647 (RM4-5)

Supplementary Fig. S2. Tissue- and peripheral-resident CD4$^+$T cells discrimination. Anti-CD4-PE-Cy7 (3µg, clone RM4-5, BD Pharmingen) was injected via tail veins of B6 (n=2) or B6.NOD-*Aec1/2* mice (n=2). After 10 minutes, mice were euthanized and salivary glands were collected and rinsed in phosphate buffered saline (PBS) for three times, 5 minutes each. Single-cell suspension was isolated as previously described [1] with digestion buffer saturated with 3µg of biotinylated anti-CD4 (clone RM4-5, BD Pharmingen). Cells were subsequently stained for anti-CD4 FITC (clone RM4-4) and Streptavidin AF-647. ®: Resident CD4$^+$ T cells, ℗: Peripheral CD4$^+$ T cells. The experiment was repeated three times for consistency. Representative experiment using a B6.NOD-*Aec1/2* mouse was presented.

1 Voigt, A. *et al.* Sexual dimorphic function of IL-17 in salivary gland dysfunction of the C57BL/6.NOD-Aec1Aec2 model of Sjogren's syndrome. *Scientific reports* **6**, 38717, doi:10.1038/srep38717 (2016).
